# A case of Eosinophilic Cellulitis causing orbital edema and ecchymosis in a child

**DOI:** 10.1016/j.ajoc.2025.102505

**Published:** 2025-12-29

**Authors:** Megan Murchison, Jeremy Maylath, Safaa Labib, Coby Ray

**Affiliations:** a Texas Tech University Health Sciences Center School of Medicine, 3601 4th Street, Lubbock, TX, 79430, USA; b Department of Ophthalmology, Texas Tech University Health Sciences Center, 3601 4th Street, Lubbock, TX, 79430, USA; c Department of Pathology, Texas Tech University Health Sciences Center, 3601 4th Street, Lubbock, TX, 79430, USA

**Keywords:** Cellulitis, Eosinophilic cellulitis, Orbit, Wells' syndrome

## Abstract

**Purpose:**

To describe an unusual case of Eosinophilic Cellulitis (Wells’ Syndrome) with orbital involvement presenting in a 10-year-old boy, which was successfully treated with systemic corticosteroids after incisional biopsy and pathologic diagnosis.

**Observations:**

A 10-year-old boy was referred to our emergency department with a sudden onset of painless ecchymosis and edema of his left periorbital region. Computed tomography was concerning for orbital cellulitis, however, the history and clinical presentation were inconsistent with this radiographic diagnosis. The patient underwent an incisional biopsy with pathology revealing eosinophilic cellulitis. The patient's condition resolved after treatment with oral corticosteroids.

**Conclusion:**

Eosinophilic cellulitis is a rare dermatosis characterized by large, inflamed, edematous patches. It usually affects adults and is mainly located on the extremities. This is a unique case due to the patient demographics and the location of this disease, with only a few orbital eosinophilic cellulitis cases reported in the literature.

## 1 Introduction

Eosinophilic cellulitis, also known as Wells’ Syndrome, is characterized by large, inflamed, edematous patches that are usually covered by vesicles or bullae and typically present on the extremities or trunk. This disease is a rare dermatosis, with the majority of reported cases affecting adults.^1^ It can be easily mistaken for bacterial cellulitis or other inflammatory dermatoses. While the exact etiology is unknown, trigger factors like insect bites, medications, infections, malignant tumors, or myeloproliferative disorders are reported.^2^

Well's Syndrome is a rare inflammatory condition that only occasionally involves the orbital or periorbital region, with few reported cases showing atypical and vision-threatening presentations. Prior reports describe adult patients with features such as unilateral ptosis or periorbital edema leading to acute vision loss, emphasizing the importance of early diagnosis and treatment.^3^^,^^4^ Our case adds to the limited literature by presenting a pediatric patient with lower eyelid swelling and bruising, an unusual presentation in both age and appearance. To the authors' knowledge, this is the first reported case of periorbital eosinophilic cellulitis in a child. We aim to highlight the importance of recognizing this rare presentation and discuss the clinical course and treatment.

## 2 Case report

**Fig. 1:**
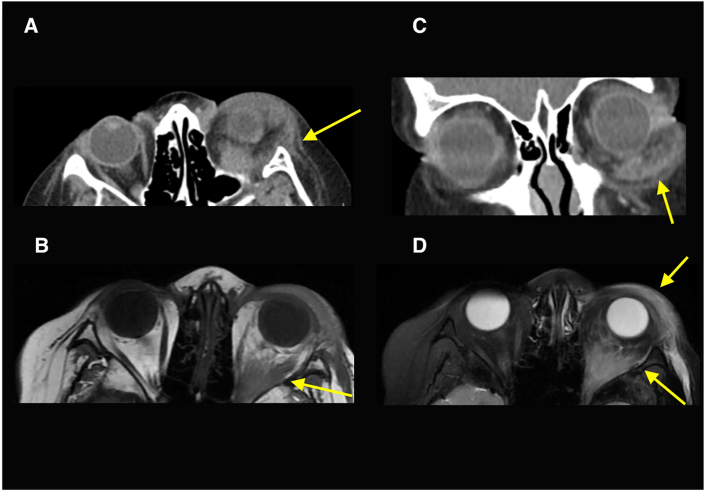
Axial and coronal CT orbits with contrast demonstrating periorbital soft tissue swelling with evidence of post-septal extension, consistent with orbital cellulitis (A,B). MRI orbits obtained the next day demonstrates similar orbital and periorbital inflammatory changes, but also notes thickening and enhancement of the inferior and lateral rectus muscles (C,D).

**Fig. 2:**
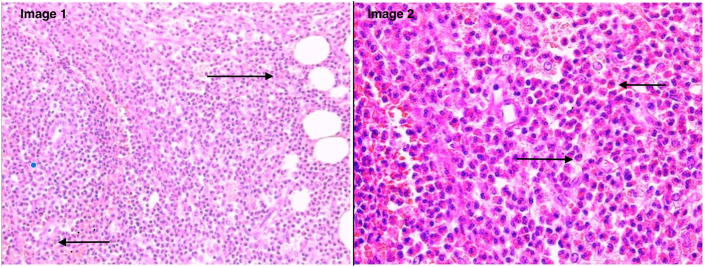
**Image 1:** High-power histologic image showing eosinophilic infiltrate. Black arrows indicate eosinophils within the infiltrate. **Image 2:** Low-power histologic image showing eosinophils infiltrating adipose tissue. Black arrows indicate eosinophils within the infiltrate.

A 10-year-old male presented to the emergency department with one week of sudden-onset, painless bruising and swelling of the left lower eyelid. He reported no trauma and had no significant past medical history. His visual acuity at presentation was 20/20 OD and 20/20 OS with intraocular pressures (IOP) of 16 OD and 21 OS. He was noted to have edema and ecchymosis of his left lower eyelid, with a palpable firm mass present in the eyelid. He was also noted to have a subconjunctival mass with a yellow appearance spanning from 2:30 o'clock to 5:30 o'clock position in the inferotemporal quadrant of the left eye. Computed tomography of the orbits was obtained, which demonstrated left periorbital soft tissue swelling and evidence of associated post-septal extension of this inflammation (Fig. 1A and B). As the history and examination were concerning for an orbital malignancy, and the radiographic features were concerning for orbital cellulitis, the patient was admitted, started on IV Clindamycin, and he underwent magnetic resonance imaging (MRI) of the orbits. The MRI showed significant inflammatory changes within the left pre-septal and post-septal soft tissues. It also demonstrated edema and enhancement of the inferior rectus muscle, lateral rectus muscle, and the lacrimal gland. Differential diagnosis based on the imaging characteristics included nonspecific orbital inflammation or orbital cellulitis. Laboratory workup was notable for eosinophilia (11.7 %; ref 0.7 %–7.0 %) and an elevated ESR (29; ref 0–15), but was otherwise negative, including CBC and CRP. Due to the possible concern of lymphoma, the oncology team ordered a skeletal survey and UA, which were both unremarkable. The patient was seen in consultation with the oculoplastics service, and it was again felt that the patient's clinic presentation was more consistent with a mass lesion than an infectious cellulitis. The patient, therefore, underwent urgent orbital and conjunctival biopsy (see Fig. 2).

Post-operatively, the patient was admitted for brief observation and the initiation of IV methylprednisolone therapy to manage his post-operative orbital and periorbital edema. He was re-evaluated in the oculoplastics clinic one day post-operatively and was found to have stable vision and IOP. He was transitioned to 40 mg oral prednisone with instructions for a brief prednisone taper, and he was discharged home to await pathologic diagnosis.

Initial pathologic evaluation was inconclusive, though the biopsy tissue was noted to contain an atypical eosinophilic infiltrate with ill-defined granulomas. The initial pathologic differential diagnosis included an infectious or allergic process, Langerhans cell histiocytosis, and orbital eosinophilic granuloma. The specimens were evaluated in consultation with the ocular pathology service at the Mayo Clinic. The tissue was noted to have an extensive acute inflammatory infiltrate with numerous eosinophils. No evidence of vasculitis or neoplasm was found. Immunostains were performed for hematopoietic and lymphoid neoplasms, but these were negative. No microorganisms were identified in the sample. The pathologic findings were felt to be most consistent with eosinophilic cellulitis.

The patient continued to follow up in the oculoplastics clinic over the next several months. He initially did well, with marked improvement in the periorbital edema on oral steroids. However, at approximately 12 weeks post-operatively, the patient experienced recurrence of his periorbital edema as well as some mild periorbital pain. He was put back on 40 mg oral prednisone, in this case, on an extended taper with a 2-week interval tapering regimen for 8 weeks. After this episode of recurrence, he was referred to pediatric rheumatology to consider steroid-sparing therapy to reduce steroid-related side effects and prevent further recurrence. He was followed in our clinic monthly for six months, but steroid-sparing therapy was not started during that time. However, at six months after biopsy, he had no further signs of periorbital or orbital inflammation off of medical therapy. After symptom resolution, he was followed every 6 months or earlier if needed.

## 3 Discussion

Eosinophilic cellulitis is a rare disease that was first described in 1971 as recurrent granulomatous dermatitis with eosinophilia.^5^ This disease often develops alongside other eosinophilic diseases, such as Churg-Strauss syndrome or cutaneous eosinophilic vasculitis.^6^ It presents with large erythematous and edematous patches with tender urticarial plaques, vesicles, bullae, or nodules. While many triggering factors, like insect bites, infections, drugs, or vaccines have been described, the pathogenesis of eosinophilic cellulitis is not well known.^6^ Eosinophilic cellulitis has been associated with hematologic malignancies or Churg-Strauss syndrome.^7^^,^^8^

The differential diagnosis for eosinophilic cellulitis is broad, and it is often initially misdiagnosed as bacterial cellulitis. If the patient does not respond to antibiotic therapy, other non-bacterial causes should be considered. Differentials can be grouped into several categories. Infectious causes include infections such as Tinea Corporis, Toxocariasis, Scabies, or erythema migrans.^6^ These infections can present with erythematous, inflamed plaques that could be mistaken for cellulitis, particularly if involving the periorbital area. Autoimmune conditions, like bullous pemphigoid or pemphigoid gestations, can involve periorbital skin and mimic cellulitis with erythematous plaques. Drug reactions or hyper-eosinophilic syndromes are also important to consider, as they can cause localized inflammation that could be mistaken for infection in the orbital area.^6^ Diagnosis is typically made with biopsy. Histopathology initially shows significant edema and a dermal infiltrate of eosinophils. Flame figures, which are composed of a central core of collagen fibers and eosinophilic granules surrounded by a histiocytic and eosinophilic infiltrate, are located in the mid to deep dermis.^1^ Eosinophilic cellulitis is often recurrent, with the period between recurrences varying from a few months to several years.^1^ However, prognosis remains good.

The main treatment options for eosinophilic cellulitis are corticosteroids and dapsone. Corticosteroids are used for all reactive dermatoses and reduce the duration of relapses.^2^ Low-dose prednisone has been recommended for patients with recurrent episodes.^6^

Dapsone is an alternative treatment and can be used alone or in conjunction with corticosteroids.^8^ Antihistamines, such as hydroxyzine, can be used for associated pruritus. Resolution rates are highest with oral steroids (92 %), followed by topical corticosteroids (50 %), and antihistamines (25 %).^2^

Due to the fact that IL-5 stimulates eosinophil production, IL-5 inhibitors have been proposed as a treatment option for eosinophilic cellulitis.^6^ One case shows effective management with mepolizumab.^8^ However, there is limited evidence to support this therapy for the treatment of eosinophilic cellulitis and this area bears further investigation.

Only a limited number of case reports document orbital and periorbital involvement and tend to exhibit atypical clinical features. In one case, an adult patient presented with unilateral ptosis, and the diagnosis was confirmed through biopsy and histological examination, revealing characteristic flame figures.^4^ The patient responded well to corticosteroid therapy, highlighting the effectiveness of early and targeted treatment.^4^ Another case report described significant periorbital edema and erythema that led to acute vision loss and elevated intraocular pressure.^3^ This case demonstrated that when eosinophilic cellulitis involves periocular tissues, it can result in serious ocular complications.^3^

In contrast, our case involves a child who presented with swelling and bruising of his left lower eyelid. This differs from previously reported cases both in terms of patient age and clinical presentation.^1^, ^2^, ^3^, ^4^ While previous reports have emphasized atypical features and potential severity of periorbital involvement, this case highlights the importance of recognizing the broader clinical spectrum of eosinophilic cellulitis, particularly in pediatric patients.^3^ Children may have difficulty communicating symptoms such as vision changes or discomfort, which increases the risk of the disease being overlooked. Additionally, the anatomical proximity of the periorbital tissue to the developing eye and brain means that inflammation in the region can progress rapidly and have more severe or lasting consequences in children.

Eosinophilic cellulitis clinically presents similarly to pre-septal or orbital cellulitis and can be difficult to differentiate.^5^ Eosinophilic cellulitis should be suspected in patients who present with peri-orbital erythematous patches, accompanied by MRI findings suggestive of an inflammatory process, such as cellulitis, but do not respond to antibiotic therapy. Recommended initial lab workup includes a CBC with differential, CMP, ESR, and CRP. A CBC showing eosinophilic predominance may suggest a diagnosis of eosinophilic cellulitis, while elevated ESR and CRP levels can indicate underlying inflammation.^1^^,^^3^ In cases where the initial biopsy raises concern for lymphoma, a skeletal survey and UA are also recommended. A skeletal survey can help identify lytic or sclerotic bone lesions suggestive of malignancy, and a UA may detect hematuria or other renal abnormalities that could be associated with systemic involvement, such as lymphoma. Biopsy showing dermal infiltrate of eosinophils and flame figures is necessary for definitive diagnosis, as bacterial cellulitis typically shows a neutrophil-predominant infiltrate.^1^^,^^3^ Recognizing eosinophilic cellulitis in the orbital or pre-orbital region is crucial for ophthalmologists, as misdiagnosis can lead to unnecessary exposure to antibiotics and corticosteroids. When this condition is overlooked or misidentified, treatment may be delayed, leading to prolonged disease despite a favorable prognosis with proper treatment.

Orbital eosinophilic cellulitis is a rare condition, particularly in the pediatric population, where available data on diagnosis and treatment remain limited. While this case demonstrates successful resolution with corticosteroid therapy, further research is essential to better understand the disease's presentation in children and to develop strategies for earlier identification and intervention to ultimately improve clinical outcomes.

## 4 Conclusion

Eosinophilic cellulitis should be considered in patients who present with signs and symptoms of cellulitis but do not respond to antibiotic therapy. This case demonstrates orbital eosinophilic cellulitis in a child, which is an unusual anatomic location and demographic. There is no standardized therapeutic approach, however eosinophilic cellulitis is very responsive to systemic steroids, but may recur following cessation of therapy.

## CRediT authorship contribution statement

**Megan Murchison:** Writing – review & editing, Writing – original draft, Investigation, Data curation. **Jeremy Maylath:** Writing – review & editing, Investigation. **Safaa Labib:** Investigation, Data curation. **Coby Ray:** Supervision, Project administration, Methodology, Conceptualization.

## Patient consent

Written consent to publish this case has not been obtained. This report does not contain any personal identifying information.

## Authorship

All authors attest that they meet the current ICMJE criteria for Authorship.

## Funding sources

No funding or grant support

## Declaration of competing interest

The authors declare that they have no known competing financial interests or personal relationships that could have appeared to influence the work reported in this paper.
